# Pre-hospital tranexamic acid administration in patients with a severe hemorrhage: an evaluation after the implementation of tranexamic acid administration in the Dutch pre-hospital protocol

**DOI:** 10.1007/s00068-023-02262-4

**Published:** 2023-04-17

**Authors:** Max Gulickx, Robin D. Lokerman, Job F. Waalwijk, Bert Dercksen, Karlijn J. P. van Wessem, Rinske M. Tuinema, Luke P. H. Leenen, Mark van Heijl

**Affiliations:** 1 Department of Surgery, University Medical Center Utrecht, C04.332, Heidelberglaan 100, 3584 CX Utrecht, The Netherlands; 2 Department of Surgery, Maastricht University Medical Center, Maastricht, The Netherlands; 3 Department of Anesthesiology, University Medical Center Groningen, Groningen, The Netherlands; 4 Regional Ambulance Facilities Utrecht, Bilthoven, The Netherlands; 5 Department of Emergency Medicine, Diakonessenhuis Utrecht/Zeist/Doorn, Utrecht, The Netherlands; 6 Trauma Center Utrecht, Utrecht, The Netherlands; 7 Department of Surgery, DiakonessenhuisUtrecht/Zeist/Doorn, Utrecht, The Netherlands

**Keywords:** Tranexamic acid, TXA, Traumatic hemorrhage, Pre-hospital, EMS professionals, Ambulance

## Abstract

**Purpose:**

To evaluate the pre-hospital administration of tranexamic acid in ambulance-treated trauma patients with a severe hemorrhage after the implementation of tranexamic acid administration in the Dutch pre-hospital protocol.

**Methods:**

All patients with a severe hemorrhage who were treated and conveyed by EMS professionals between January 2015, and December 2017, to any trauma-receiving emergency department in the eight participating trauma regions in the Netherlands, were included. A severe hemorrhage was defined as extracranial injury with > 20% body volume blood loss, an extremity amputation above the wrist or ankle, or a grade ≥ 4 visceral organ injury. The main outcome was to determine the proportion of patients with a severe hemorrhage who received pre-hospital treatment with tranexamic acid. A Generalized Linear Model (GLM) was performed to investigate the relationship between pre-hospital tranexamic acid treatment and 24 h mortality.

**Results:**

A total of 477 patients had a severe hemorrhage, of whom 124 patients (26.0%) received tranexamic acid before arriving at the hospital. More than half (58.4%) of the untreated patients were suspected of a severe hemorrhage by EMS professionals. Patients treated with tranexamic acid had a significantly lower risk on 24 h mortality than untreated patients (OR 0.43 [95% CI 0.19–0.97]).

**Conclusion:**

Approximately a quarter of the patients with a severe hemorrhage received tranexamic acid before arriving at the hospital, while a severe hemorrhage was suspected in more than half of the non-treated patients. Severely hemorrhaging patients treated with tranexamic acid before arrival at the hospital had a lower risk to die within 24 h after injury.

**Supplementary Information:**

The online version contains supplementary material available at 10.1007/s00068-023-02262-4.

## Background

Traumatic injuries are responsible for one out of ten deaths worldwide, and approximately one-third of these patients die as a result of hemorrhage [1, 2]. Tranexamic acid is an antifibrinolytic agent which reduces mortality in hemorrhaging trauma patients, without increasing their chance of vascular-occlusive events [3, 4]. The improvement of survival is greatest when tranexamic acid is administered as soon as possible after injury [5–7]. Therefore, current clinical guidelines recommend administering the first dose of tranexamic acid at the scene of injury or en route to the hospital to trauma patients whom are hemorrhaging or at risk of a significant hemorrhage [8, 9].

In most inclusive trauma systems, pre-hospital treatment is generally performed by Emergency Medical Services (EMS) professionals, and the use of tranexamic acid has been widely adopted in their pre-hospital protocols over the past decade. Administrating tranexamic acid was incorporated in the Dutch National Protocol of Ambulance Services (NPAS) in 2014. Since then, Dutch EMS professionals are advised to administer tranexamic acid to patients with a hypovolemic shock due to an uncontrollable hemorrhage [10]. A previous study, performed in the United Kingdom, found that many hemorrhaging patients were not adequately treated with tranexamic acid after incorporating its use in their pre-hospital and in-hospital protocols [11]. However, pre-hospital treatment rates could not be determined as the proportion of patients treated before arriving at the hospital was not described.

It remains unclear to what extent patients who could benefit from tranexamic acid are accurately treated before arrival at the hospital. This study, therefore, aims to determine the proportion of ambulance-transported trauma patients with a severe hemorrhage who are treated with tranexamic acid and investigates the possible causes of non-treatment.

## Methods

The current study was reported in line with the STROBE guidelines [12]. The Medical Ethical Committee of the University Medical Center Utrecht decided that the Medical Research Involving Human Subjects Act did not apply to this study (reference number: 20/500747).

### Study setting

In this study, seven ambulance services and eight inclusive trauma regions participated in the data collection. Annually, around 550,000 patients are transported to a hospital by the participating ambulance services [13]. They serve a region of approximately 8000km^2^ that contains more than 6.5 million residents. The participating trauma regions comprise eight higher-level and 60 lower-level trauma centers. In the Netherlands, inclusive trauma regions contain at least one higher-level trauma center (i.e., level-I trauma center), specialized in treating severely injured patients, and multiple lower-level trauma centers (i.e., level-II and level-III trauma centers), designed to treat mildly and moderately injured patients [14]. Higher-level trauma centers in the Netherlands adhere to the standards of the American College of Surgeons Committee on Trauma (ACSCOT) for providing the highest level of trauma care [15].

Dutch ambulances are staffed by an EMS professional (i.e., specialized registered nurse) licensed to provide advanced life trauma care, and a qualified ambulance-driver able to provide medical assistance. Since 2014, the 8^th^ version of the Dutch NPAS is used which recommends administering tranexamic acid to patients with a hypovolemic shock (systolic blood pressure < 90 mmHg) due to an uncontrollable hemorrhage [10]. Contra-indications to administer tranexamic acid according to the Dutch NPAS are: active thrombo-embolic events, > 3 h after the moment of injury, and age < 1 year. Before tranexamic acid was introduced in the Dutch NPAS, EMS professionals were trained and tested on the (contra) indications of administering tranexamic acid and the diagnostic limitation of recognizing severe hemorrhage was discussed. In the Netherlands, the Helicopter Emergency Medical Service (HEMS) can be dispatched to assist the EMS professionals at the scene of injury or during transportation to the hospital if a patient is unstable or expected to become unstable and is staffed by a specialized physician (i.e., trauma surgeon or emergency anesthesiologist). Due to the relatively short transportation distances in the Netherlands, only few patients are transported by helicopter. The Dutch HEMS uses the same criteria to administer tranexamic acid as described in the Dutch NPAS.

### Patients

Patients with a severe hemorrhage, who were transported by a ground ambulance of one of the seven participating ambulance services to any trauma-receiving emergency department in the eight participating trauma regions, were included.

### Data collection

Records of all patients transported by a ground ambulance of one of the seven participating ambulance services between January 1st, 2015, and December 31st, 2017, were prospectively collected. Non-trauma patients, patients transported to a non-participating trauma region, patients without the in-hospital diagnosis of a severe hemorrhage, and patients with a contra-indication to receive tranexamic acid, were excluded. Trauma patients were identified from unfiltered EMS records using a previously developed selection tool with an accuracy of 98.9% (95%-CI, 98.3–99.2) [16]. The pre-hospital patient records were written by EMS professionals and contained the patient’s demographics, pre-hospital vital signs, pre-hospital injury suspicion, tranexamic acid administration, HEMS assistance, and transportation details (e.g., transport destination). Pre-hospital patient records were assessed to determine if the EMS professional had a suspicion of a severe hemorrhage at the scene of injury and whether the patient had a contra-indication to receive tranexamic acid. Qualified data managers of the regional trauma registries collected injury mechanisms, in-hospital resource use, mortality status (all-cause), and designated AIS codes based on in-hospital diagnosed injuries, of all admitted patients. Pre-hospital and in-hospital patient records were linked with an externally validated linkage tool with an accuracy of 100.0% (95%-CI, 100.0–100.0) [16].

### Outcomes and definitions

The main outcome of this study was to determine the proportion of ambulance-transported patients with a severe hemorrhage who were treated with tranexamic acid before arrival at the hospital. A severe hemorrhage was defined as an in-hospital diagnosed extracranial injury with AIS codes including the following descriptions: > 20% body volume blood loss, extremity amputation above the wrist or ankle, or grade ≥ 4 visceral organ injuries (Online Appendix 1) [17]. Patients were suspected to have a severe hemorrhage at the scene of injury if the EMS professionals described a substantial external hemorrhage (i.e., estimated blood loss ≥ 1000 ml) or the suspicion of an internal hemorrhage in the pre-hospital records, or if the patient had a systolic blood pressure < 90 mmHg at the scene of injury.

### Missing data

Variables with missing values were systolic blood pressure (23.8%), heart rate (10.7%), respiratory rate (28.3%), Glasgow Coma Scale (10.7%), and total pre-hospital times (6.7%) and appeared to be missing at random. These variables were multiply imputed using, amongst others, patients’ characteristics, pre-hospital vital signs, and in-hospital outcomes, in a multilevel multiple imputation creating 48 imputed datasets based on 20 iterations per set.

### Statistical analysis

Descriptive statistics were used to analyze the data, and the results are presented in frequencies and percentages or median and inter-quartile range (IQR). The *χ*^2^ or Mann–Whitney *U* test were used to compare groups and we considered p values < 0.05 as statistically significant. The results of the multiply imputed variables were pooled from each imputed data set following Rubin’s rules [18]. A Generalized Linear Model was performed to investigate the relationship between tranexamic acid and 24 h mortality, adjusted for age, gender, injury severity (i.e., ISS), penetrating injury, HEMS assistance, distance to level-1 trauma center, and vital parameters used in the Revised Trauma Score (RTS) (i.e., systolic blood pressure, respiratory rate, and Glasgow Coma Scale). We used restricted cubic splines to adjust for non-linearity. All statistical analyses were computed with R statistical software (R version 4.0.3) [19].

## Results

**Fig. 1:**
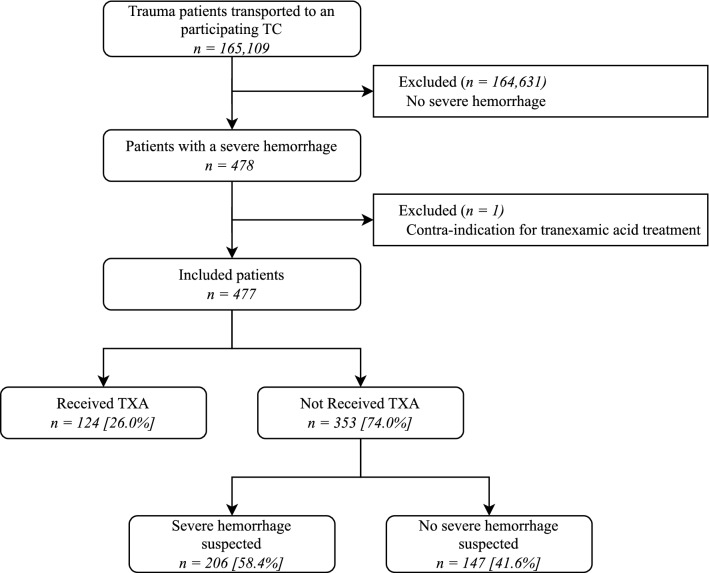
Patient selection. *TC* trauma center, *TXA* tranexamic acid

A total of 165,109 trauma patients were transported to trauma-receiving emergency departments in the eight participating trauma regions during the study period, of whom 3760 patients (2.3%) were severely injured (Injury Severity Score [ISS] ≥ 16) (Fig. 1). In total, 478 patients had a severe hemorrhage, and after excluding one patient who had a contra-indication to receive tranexamic acid, 477 patients were included.

Baseline characteristics are shown in Table 1. The median age of the patients with a severe hemorrhage was 40.7 (IQR, 25.6–57.2) years, 335 (70.2%) patients were male, and 346 (72.5%) patients were severely injured (ISS ≥ 16). Fifty-five (11.5%) patients had a systolic blood pressure < 90 mmHg at the scene of injury, 146 (30.6%) patients had a penetrating injury, and 51 (10.7%) and 84 (17.6%) patients died within 24 h and 30-days, respectively.

**Table 1:** Baseline characteristics

Variables	All patients with a severe hemorrhage *n* = 477	Tranexamic acid + *n* = 124	Tranexamic acid − *n* = 353	*p* value
Demographics	Median (IQR)	Median (IQR)	Median (IQR)	
Age (years)	40.7 (25.6–57.2)	36.1 (25.2–53.8)	42.7 (25.8–59.9)	0.160
	*N* (%)	*N* (%)	*N* (%)	
Age < 16 (years)	20 (4.2)	3 (2.4)	17 (4.8)	0.376
Age ≥ 65 (years)	91 (19.1)	19 (15.3)	72 (20.4)	0.270
Male gender	335 (70.2)	97 (78.2)	238 (67.4)	0.032
ISS, median (IQR)	21 (13–34)	28 (17–41)	18 (13–29)	< 0.001
ISS ≥ 16	346 (72.5)	102 (82.3)	244 (69.1)	0.007
Type of trauma	*N* (%)	*N* (%)	*N* (%)	
Private	126 (26.4)	17 (13.7)	109 (30.9)	< 0.001
Traffic	185 (38.8)	53 (42.7)	132 (37.4)	0.345
Sport	16 (3.4)	1 (0.8)	15 (4.2)	0.082*
Industrial	39 (8.2)	13 (10.5)	26 (7.4)	0.368
Violence	57 (11.9)	25 (20.2)	33 (9.3)	0.005
Self-mutilation	48 (10.1)	14 (11.3)	38 (10.1)	0.723
Different	2 (0.4)	1 (0.8)	1 (0.3)	0.453*
Vital parameters	*N* (%)	*N* (%)	*N* (%)	
Systolic blood pressure < 90 mmHg	55 (11.5)	24 (19.4)	31 (8.8)	0.003
Heart rate > 110 bpm	106 (22.2)	46 (37.1)	60 (17.0)	< 0.001
Respiratory rate > 29/min or < 10/min	48 (10.1)	20 (16.1)	28 (7.9)	0.015
Glasgow Coma Scale score < 13	115 (24.1)	52 (41.9)	63 (17.8)	< 0.001
Revised Trauma Score < 12	170 (35.6)	72 (58.1)	98 (27.8)	< 0.001
Pre-hospital assessment	*N* (%)	*N* (%)	*N* (%)	
Penetrating injury	146 (30.6)	44 (35.5)	102 (28.9)	0.209
Stab wound	101 (21.2)	33 (26.6)	68 (19.3)	0.111
Gunshot wound	19 (4.0)	7 (5.6)	12 (3.4)	0.289*
Suspected hemorrhage	330 (69.2)	124 (100.0)	206 (58.4)	< 0.001
HEMS assistance	199 (41.7)	94 (75.8)	105 (29.7)	< 0.001
Transportation characteristics	*N* (%)	*N* (%)	*N* (%)	
Transported to a higher-level trauma center	379 (79.5)	115 (92.7)	264 (74.8)	< 0.001
	Median (IQR)	Median (IQR)	Median (IQR)	
Response time, min	7.7 (5.4–10.2)	6.9 (5.0–9.3)	8.0 (5.3–10.7)	0.024
On-scene time, min	20.9 (13.9–29.2)	23.9 (16.7–31.2)	20.0 (13.0–28.1)	0.009
Transport time, min	12.6 (7.1–20.6)	14.5 (8.6–27.4)	12.3 (7.0–19.0)	0.004
Total pre-hospital time, min	43.7 (33.0–58.2)	50.4 (38.5–64.7)	42.0 (32.0–55.4)	< 0.001
Intervention and outcomes	*N* (%)	*N* (%)	N (%)	
Emergency intervention**	154 (32.3)	51 (41.1)	103 (29.2)	0.019
24 h mortality	51 (10.7)	16 (12.9)	35 (9.9)	0.449
30-day mortality	84 (17.6)	32 (25.8)	52 (14.7)	0.008

*ISS* Injury Severity Score; *IQR*, Interquartile range. Systolic blood pressure missed in 23.3%, heart rate in 12.8%, respiratory rate in 28.3%, Glasgow Coma Scale in 10.7%, and total pre-hospital time in 6.7% of the patients

^*^Fisher’s exact test

^**^Emergency intervention: damage control orthopedics, Damage control laparotomy, Damage control thoracotomy, ICP monitoring, Craniotomy, Extremity revascularization, Extraperitoneal pelvic packaging

### Treatment rates

**Fig. 2:**
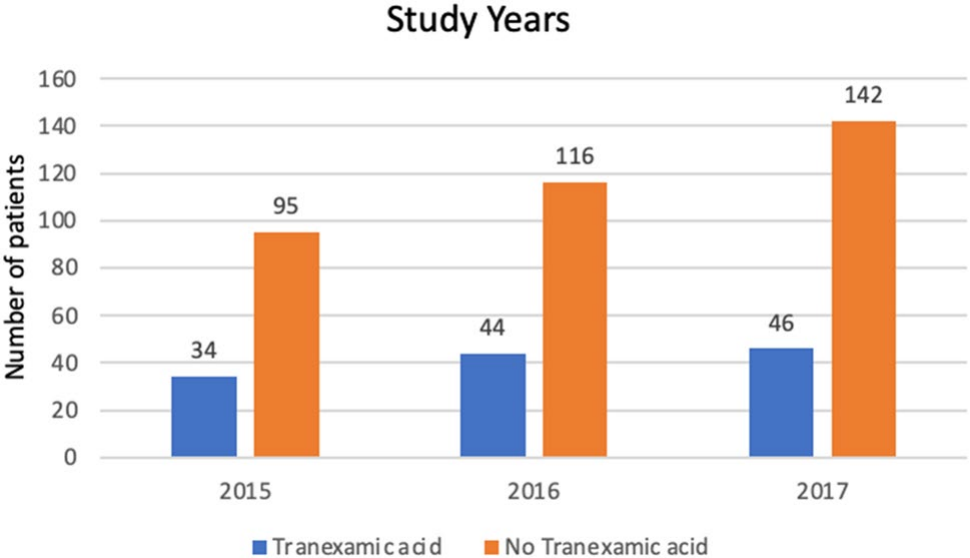
Tranexamic acid treatment during study period

In total, 124 (26.0%) patients with a severe hemorrhage were treated with tranexamic acid before arrival at an emergency department. The HEMS assisted in 199 patients, of which 94 (47.2%) were treated with tranexamic acid. Treatment rates did not significantly change during the study period (26.9% in 2015, 27.5% in 2016, and 24.5% in 2017) (Fig. 2). Treatment rates ranged between 11.3% and 50.0% in the seven participating ambulance services (Online Appendix 2), and sensitivity analyses for patients without missing SBP or isolated grade ≥ 4 visceral organ injury showed treatment rates of 25.7% and 29.2%, respectively (Online Appendix 3).

### Treated patients

Patients who were treated with tranexamic acid were younger (median age [IQR], 36.1 [25.2–53.8] vs. 42.7 [25.8–59.9]; *p* 0.160), more often male (97 [78.2%] vs. 238 [67.4%]; *p* 0.032), and more severely injured (median ISS [IQR], 28 [17–41] vs. 18 [13–29]; *p* < 0.001), than the untreated patients. The treated patients also had more often penetrating injuries (44 [35.5%] vs. 102 [28.9%]; *p* 0.209), had more often a decreased (< 12) RTS (72 [58.1%] vs. 98 [27.8%]; *p* < 0.001), and an emergency intervention was more often performed in these patients (51 [41.1%] vs 103 [29.2%]; *p* < 0.019). Furthermore, the treated patients were more often transported to a higher-level trauma center (vs. 115 [92.7%] vs. 264 [74.8%]; *p* < 0.001), their total pre-hospital times were longer (median minutes [IQR], 50.4 [38.5–64.7] vs. 42.0 [32.0–55.4]; *p* < 0.001), and the HEMS assisted more often in these patients (94 [75.8%] vs. 105 [29.7%]; *p* < 0.001).

Two-hundred-six (58.4%) patients who were not treated with tranexamic acid were suspected to have a severe hemorrhage (Table 2). Thirty-seven (18.0%) of these patients had a systolic blood pressure < 90 mmHg, 122 (59.2%) patients had substantial external blood loss at the scene of injury, and 88 (42.7%) patients were suspected to have an internal hemorrhage.

**Table 2:** Suspected and unsuspected hemorrhages of non-treated patients

Variables	Tranexamic acid *n* = 353	Suspicion of a severe hemorrhage *n* = 206	No suspicion of a severe hemorrhage *n* = 147	*p* value
Demographics	Median (IQR)	Median (IQR)	Median (IQR)	
Age (years)	42.7 (25.8–59.9)	43.2 (26.1–59.1)	41.2 (25.5–60.1)	0.655
	*N* (%)	*N* (%)	*N* (%)	
Age < 16 (years)	17 (4.8)	10 (4.9)	7 (4.8)	1
Age ≥ 65 (years)	72 (20.4)	41 (19.9)	31 (21.1)	0.890
Male gender	238 (67.4)	145 (70.4)	93 (63.3)	0.196
ISS, median (IQR)	18 (13–29)	17 (10–26)	21 (16–29)	0.004
Type of trauma	*N* (%)	*N* (%)	*N* (%)	
Private	109 (30.9)	73 (35.4)	36 (24.5)	0.038
Traffic	132 (37.4)	57 (27.7)	75 (51.0)	< 0.001
Sport	15 (4.2)	8 (3.9)	7 (4.8)	0.892
Industrial	26 (7.4)	14 (6.8)	12 (8.2)	0.999
Violence	33 (9.3)	26 (12.6)	7 (4.8)	0.021
Self-mutilation	38 (10.1)	25 (12.1)	9 (6.1)	0.088
Different	1 (0.3)	1 (0.5)	0 (0.0)	1
Vital parameters	*N* (%)	*N* (%)	*N* (%)	
Systolic blood pressure < 90 mmHg	37 (10.5)	37 (18.0)	Na	< 0.001
Heart rate > 110 bpm	67 (19.0)	46 (22.3)	21 (14.3)	0.062
Respiratory rate > 29/min or < 10/min	36 (10.2)	26 (12.6)	10 (6.8)	0.097
Glasgow Coma Scale score < 13	77 (21.8)	53 (25.7)	24 (16.3)	0.138
Revised Trauma Score < 12	116 (32.9)	88 (42.7)	28 (19.0)	< 0.001
Pre-hospital assessments	*N* (%)	*N* (%)	*N* (%)	
Penetrating injury	102 (28.9)	93 (45.1)	9 (6.1)	< 0.001
Described external hemorrhage*	122 (34.6)	122 (59.2)	Na	< 0.001
Suspected internal hemorrhage*	88 (24.9)	88 (42.7)	Na	< 0.001
HEMS assistance	105 (29.7)	60 (29.1)	45 (30.6)	0.855
Regions involved*	*N* (%)	*N* (%)	*N* (%)	
Head, face, or neck	59 (16.7)	44 (21.4)	15 (10.2)	0.009
Internal hemorrhage	207 (58.6)	95 (46.1)	112 (76.2)	< 0.001
Thoracic	51 (13.5)	29 (14.1)	19 (12.9)	0.878
Abdominal	154 (40.7)	59 (28.6)	87 (59.2)	< 0.001
Pelvic	16 (4.5)	9 (4.4)	7 (4.8)	1
Extremities	107 (30.3)	78 (37.9)	29 (19.7)	< 0.001
Transportation characteristics	*N* (%)	*N* (%)	*N* (%)	
Transported to a higher-level trauma center	264 (74.8)	157 (76.2)	107 (72.8)	0.544
Pre-hospital treatment time** > 15 min	316 (94.3)	185 (93.9)	131 (94.9)	0.875
Injuries and outcomes	*N* (%)	*N* (%)	*N* (%)	
Emergency intervention***	103 (29.2)	68 (33.0)	35 (23.8)	0.079
24 h mortality	35 (9.9)	26 (12.6)	9 (6.1)	0.067
30-day mortality	52 (14.7)	37 (18.0)	15 (10.2)	0.061

*ISS* Injury Severity Score; *IQR* Interquartile range

All missing values were multiply imputed

^*^Patients can sustain severe hemorrhages in multiple body regions

^**^Pre-hospital treatment time: treatment time at the scene of injury + transportation time

^***^Emergency intervention: Damage control orthopedics, Damage control laparotomy, Damage control thoracotomy, ICP monitoring, Craniotomy, Extremity revascularization, Extraperitoneal pelvic packaging

Patients treated with tranexamic acid had significantly lower risk on 24 h mortality than untreated patients (OR 0.43 [95%CI, 0.19–0.97]) (Table 3).

**Table 3:** Adjusted odds ratio for 24 h mortality

Pre-hospital tranexamic acid	Generalized linear model
	Odds ratio (95% CI)
No tranexamic acid	Reference
Tranexamic acid + *	0.43 (0.19–0.97)

^*^Adjusted for: age, gender, injury severity (ISS), penetrating injury, HEMS assistance, distance to level-1 trauma center, systolic blood pressure, Glasgow coma scale, and respiratory rate

## Discussion

This study evaluated pre-hospital tranexamic acid administration in patients with a severe hemorrhage and found that 26% of these patients were treated with tranexamic acid before arrival at the hospital while more than half (58%) of the untreated patients were suspected to have a severe hemorrhage. Additionally, we found that patients who were treated with tranexamic acid before arrival at the hospital had significantly lower risk on 24 h mortality than untreated patients (adjusted OR 0.44 [95% CI, 0.19–0.97]).

This was, to our knowledge, the first trauma-region wide study that was able to adequately estimate the pre-hospital tranexamic acid administration rate in severely hemorrhaging patients. We found that a majority of these patients were not treated before arrival at the hospital, which is in line with the findings of previous studies [11, 20]. Coats et al. described the implementation of tranexamic acid in the pre-hospital and in-hospital protocols in England and Wales between 2010 and 2016 and found low administration rates in the TARN database (10%), even in patients with a systolic blood pressure < 100 mmHg at the emergency department (23.8%) [11]. This study, however, did not describe the proportion of patients that were treated before arrival at the hospital. Another study by Van Wessem et al. found that 49% of the severely injured patients who were admitted to a Dutch Level-I trauma center received tranexamic acid before arrival at the hospital [20]. In the current study, we found lower treatment rates probably due to the fact that we also included patients with a severe hemorrhage who were transported to a lower-level trauma center and/or were not severely injured (ISS < 16).

Accurately recognizing a severe hemorrhage at the scene of injury can be challenging as it is difficult to estimate the amount of external blood loss [21], internal hemorrhages can be difficult to recognize [22–24], and patients can become hypotensive at a later stage or due to other causes (e.g., neurogenic shock) [25, 26]. In the current study, solely a small proportion of the patients with a severe hemorrhage had a systolic blood pressure < 90 mmHg. This could suggest that, in a highly developed trauma system with short transportation distances, blood pressure might not be the right parameter to estimate if a patient is severely hemorrhaging at the scene of injury. The advice to administer tranexamic acid solely in patients with a hypovolemic shock due to an uncontrollable hemorrhage, as prescribed in the Dutch NPAS [10], might have caused the EMS professional to leave these patients untreated as more than half (58%) of the non-treated patients were suspected to have a severe hemorrhage. Moreover, the relatively short distances in the Netherlands, and the shorter total pre-hospital times in patients that did not receive tranexamic acid (median minutes, 42 vs. 50), might have caused the EMS professionals to postpone tranexamic acid administration until arrival at an emergency department in the patients they suspected to have a severe hemorrhage. However, every 15 min of treatment delay decreases the survival benefit of tranexamic acid by 10% [7], which indicates that 94% of the untreated patients could have benefited from pre-hospital tranexamic acid administration as they had a pre-hospital time longer than 15 min.

### Strengths and limitations

The inclusion of patients from a large, consecutive cohort, is a great strength of this study. Pre-hospital records of patients transported by seven ambulance services, which serve rural, urban, and suburban areas, were linked to their in-hospital records from both higher-level and lower-level trauma centers in the eight participating trauma regions. All admitted trauma patients are registered by the Dutch trauma registry, and in the Netherlands, no patients are transported to or treated at a non-trauma center [27]. This resulted in the inclusion of as many patients with a severe hemorrhage as possible, even if transported to an adjacent trauma region. This study also has some limitations. First, the total number of patients that could have benefitted from tranexamic acid can be greater than outlined in this study, as we solely included patients with severe traumatic hemorrhages. Second, as there are no specific AIS codes for blood loss due to visceral organ injuries, we additionally included patients with grade ≥ 4 injuries to prevent excluding patients with severe hemorrhages due to visceral organ injuries. However, a sensitivity analysis in patients without grade ≥ 4 visceral organ injuries demonstrated a comparable treatment rate of 29.1% vs. 26.0% in all patients. Third, we performed a cohort study on patients that were severely hemorrhaging and retrospectively determined if they received tranexamic acid, which might contribute to the relatively low treatment rates. Final, we were unable to adjust the association between pre-hospital treatment with tranexamic acid and 24 h mortality for in-hospital tranexamic acid treatment, as it was not recorded by the Dutch Trauma Registry.

Previous studies demonstrate the effectivity and safety of tranexamic acid in a wide spectrum of patients and indicate the importance of early administration [3, 7, 28–31]. The European guideline of major bleeding and coagulopathy following trauma therefore recommends, since 2016, to treat both hemorrhaging patients and patients at risk of a significant hemorrhage before arrival at the hospital to prevent undertreatment in patients who could have benefitted from tranexamic acid, as per the inclusion criteria of the CRASH-II study [5, 9]. Adjusting the current criteria of the Dutch NPAS for administering tranexamic acid, and alike protocols of other countries, to such a more liberal definition (i.e., administer tranexamic acid in patients with and patients suspected of a severe hemorrhage), could potentially improve pre-hospital treatment rates substantially as the majority of the patients (69%) was suspected of a severe hemorrhage by the EMS professionals. However, future research should investigate the effect of such a liberal adjustment on overtreatment and patient outcomes.

Additionally, although a large proportion of the patients with a severe hemorrhage were suspected of a severe hemorrhage by the EMS professionals, still a third of the patients remained unrecognized. Future research could focus on the development of pre-hospital prediction models to aid EMS professionals in recognizing a severe hemorrhage which could further improve pre-hospital treatment rates. Such a prediction model has been previously developed to predict if a patient is severely injured at the scene of injury [32].

## Conclusion

This study found that approximately a quarter of the patients with a severe hemorrhage received tranexamic acid before arrival at the hospital, while more than half of the untreated patients were suspected to have a severe hemorrhage by the EMS professionals. The low treatment rate seemed to be caused by the short transportation distances, recommendations in the pre-hospital protocol to solely treat the most severely hemorrhaging patients, and the challenges of recognizing a severe hemorrhage in the field. Severely hemorrhaging patients treated with tranexamic acid before arrival at the hospital had lower risk to die within 24 h after injury. We suggest revising the pre-hospital protocol to a more user-friendly version in the field to aid EMS professionals in their on-scene decision making.

## Data and code availability

The data that supports the findings of the current study is not publicly available due to its sensitive nature but is available upon a reasonable request that needs to be approved of the participating Emergency Medical Services and trauma regions, provided that appropriate ethical approval is sought. R-scripts are available upon request.

### Supplementary Information

Below is the link to the electronic supplementary material.

Supplementary file1 (DOCX 25 KB)

Supplementary file2 (DOCX 14 KB)

Supplementary file3 (DOCX 14 KB)

Supplementary file4 (DOCX 16 KB)
