# Supplementary material for: Pre-hospital tranexamic acid administration in patients with a severe hemorrhage: an evaluation after the implementation of tranexamic acid administration in the Dutch pre-hospital protocol
Source: Eur J Trauma Emerg Surg. 2023 Apr 17;50(1):139–47. doi: 10.1007/s00068-023-02262-4 (PMC10923991; doi:10.1007/s00068-023-02262-4)
Supplement: Supplementary file 1 — Supplementary file1 (DOCX 25 KB) [file 68_2023_2262_MOESM1_ESM.docx]

| **Appendix 1.** Selected AIS codes that define a significant hemorrhage | | | | | |
| --- | --- | --- | --- | --- | --- |
| **Code** | **Region** | **Injury Description** | **AIS98** | **AIS98exact** | **FCI** |
| 110606.3 | Scalp | Laceration blood loss >20% by volume | 110606.3 | 110606.3 | 5 |
| 110806.3 | Scalp | Avulsion blood loss >20% by volume | 110806.3 | 110806.3 | 5 |
| 216006.3 | Penetrating injury | With blood loss >20% by volume | 216006.3 | 216006.3 | 5 |
| 210606.3 | Skin/subcutaneous/muscle | Laceration blood loss >20% by volume | 210606.3 | 210606.3 | 5 |
| 210806.3 | Skin/subcutaneous/muscle | Avulsion blood loss >20% by volume | 210806.3 | 210806.3 | 5 |
| 220204.3 | External carotid artery branch(es) laceration | Major; transection; blood loss >20% by volume | 220204.3 | 220204.3 | 5 |
| 250810.4 | Maxilla | Fracture LeFort Ill blood loss >20% by volume | 250810.4 | 250810.4 | 5 |
| 251902.4 | Panfacial | Fracture blood loss >20% by volume | None | None | 3 |
| 316006.3 | Penetrating Injury | Blood loss >20% by volume | 316006.3 | 316006.3 | 5 |
| 310606.3 | Skin/subcutaneous tissue/muscle | Laceration blood loss >20% by volume | 310606.3 | 310606.3 | 5 |
| 310806.3 | Skin/subcutaneous tissue/muscle | Avulsion blood loss >20% by volume | 310806.3 | 310806.3 | 5 |
| 320212.4 | Carotid artery [common, internal] | Laceration; perforation; puncture major; rupture; transection; segmental loss; blood loss >20% by volume | 320212.4 | 320212.4 | 5 |
| 320213.4 | Carotid artery [common, internal] | Laceration; perforation; puncture major; rupture; transection; segmental loss; blood loss >20% by volume bilateral | 320212.4 | None | 5 |
| 320214.5 | Carotid artery [common, internal] | Laceration; perforation; puncture major; rupture; transection; segmental loss; blood loss >20% by volume neurological deficit (stroke) not head-injury related | 320214.5 | 320214.5 | 1 |
| 320215.5 | Carotid artery [common, internal] | Laceration; perforation; puncture major; rupture; transection; segmental loss; blood loss >20% by volume neurological deficit (stroke) not head-injury related bilateral | 320214.5 | None | 1 |
| 320408.3 | Carotid artery [external] | Laceration; perforation; puncture major; rupture; transection; segmental loss; blood loss >20% by volume | 320408.3 | 320408.3 | 5 |
| 321012.3 | Vertebral artery | Laceration; perforation; puncture major; rupture; transection; blood loss >20% by volume | 321012.3 | 321012.3 | 5 |
| 321014.4 | Vertebral artery | Laceration; perforation; puncture major; rupture; transection; blood loss >20% by volume neurological deficit (stroke) not head-injury related | 321014.4 | 321014.4 | 2 |
| 321015.5 | Vertebral artery | Laceration; perforation; puncture major; rupture; transection; blood loss >20% by volume neurological deficit (stroke) not head-injury related bilateral | 321014.4 | None | 1 |
| 320606.3 | Jugular vein [external] | Major; rupture; transection; segmental loss; blood loss >20% by volume | 320606.3 | 320606.3 | 5 |
| 320806.3 | Jugular vein [internal] | Laceration; perforation; puncture major; rupture; transection; blood loss >20% by volume | 320806.3 | 320806.3 | 5 |
| 416006.3 | Penetrating injury | With blood loss >20% by volume | 416006.3 | 416006.3 | 5 |
| 410606.3 | Skin/subcutaneous /muscle | Blood loss >20% by volume | 410606.3 | 410606.3 | 5 |
| 410806.3 | Skin/subcutaneous /muscle | Blood loss >20% by volume | 410806.3 | 410806.3 | 5 |
| 420210.5 | Aorta, thoracic | Major; rupture; transection; segmental loss; blood loss >20% by volume | 420210.5 | 420210.5 | 5 |
| 420408.4 | Brachiocephalic (innominate) artery | Laceration; perforation; puncture major; rupture; transection; segmental loss; blood loss >20% by volume | 420408.4 | 420408.4 | 5 |
| 421008.5 | Pulmonary artery | Laceration; perforation; puncture major; rupture; transection; segmental loss; blood loss >20% by volume | 421008.4 | 421008.4 | 5 |
| 421009.6 | Pulmonary artery | Laceration; perforation; puncture major; rupture; transection; segmental loss; blood loss >20% by volume bilateral | 421008.4 | None | 1 |
| 421408.4 | Subclavian artery | Major; rupture; transection; segmental loss; blood loss >20% by volume | 421408.4 | 421408.4 | 5 |
| 422008.3 | Other named arteries e.g., bronchial, esophageal, intercostal, internal mammary | Laceration; perforation; puncture major; rupture; transection; segmental loss; blood loss >20% by volume | 422008.3 | 422008.3 | 5 |
| 420606.4 | Brachiocephalic (innominate) vein | Laceration; perforation; puncture major; rupture; transection; segmental loss; blood loss >20% by volume | 420606.4 | 420606.4 | 5 |
| 420608.5 | Brachiocephalic (innominate) vein | Laceration; perforation; puncture major; rupture; transection; segmental loss; blood loss >20% by volume with air embolus right side | 420608.5 | 420608.5 | 5 |
| 421206.5 | Pulmonary vein | Laceration; perforation; puncture major; rupture; transection; segmental loss; blood loss >20% by volume | 421206.e | 421206.4 | 5 |
| 421207.6 | Pulmonary vein | Laceration; perforation; puncture major; rupture; transection; segmental loss; blood loss >20% by volume bilateral | 421206.4 | None | 1 |
| 421604.3 | Subclavian vein | Laceration; perforation; puncture minor; superficial; incomplete circumferential involvement; blood loss 020% by volume | 421604.3 | 421604.3 | 5 |
| 421606.4 | Subclavian vein | Laceration; perforation; puncture major; rupture; transection; segmental loss: blood loss >20% by volume | 421606.4 | 421606.4 | 5 |
| 421806.4 | Vena Cava, superior and thoracic portion of inferior, | Laceration; perforation; puncture major; rupture; transection; segmental loss; blood loss >20% by volume | 421806.4 | 421806.4 | 5 |
| 421808.5 | Vena Cava, superior and thoracic portion of inferior, | Laceration; perforation; puncture major; rupture; transection; segmental loss; blood loss >20% by volume with air embolus right side | 421808.5 | 421808.5 | 5 |
| 422206.3 | Other named veins, e.g., azygos, bronchial, esophageal, hemiazygos, intercostal, internal jugular, internal mammary | Laceration; perforation; puncture major; rupture; transection; segmental loss; blood loss >20% by volume | 422206.3 | 422206.3 | 5 |
| 441012.5 | Heart (Myocardium) injury | Laceration perforation, ventricular or atrial, with or without tamponade | 441012.5 | 441012.5 | 5 |
| 441013.5 | Heart (Myocardium) injury | Perforation atrial rupture | 441013.5 | 441013.5 | 5 |
| 441014.6 | Heart (Myocardium) injury | Perforation ventricular rupture | 441014.6 | 441014.6 | 1 |
| 441016.6 | Heart (Myocardium) injury | Perforation multiple lacerations; >50% tissue loss of a chamber | 441016.6 | 441016.6 | 1 |
| 441018.6 | Heart (Myocardium) injury | Avulsion | 441018.6 | 441018.6 | 1 |
| 442201.4 | Thoracic injury | Hemothorax major; >1000cc blood loss on at least one side | 442208.4 | 442208.4 | 5 |
| 442206.4 | Thoracic injury | Hemopneumothorax major; >1000cc blood loss on at least one side | 442202.3 | None | 5 |
| 516006.3 | Penetrating injury | With blood loss >20% by volume | 516006.3 | 516006.3 | 5 |
| 510604.2 | Skin/subcutaneous/muscle [except rectus abdominus] | Laceration major; >20cm long and into subcutaneous tissue | 510604.2 | 510604.2 | 5 |
| 510806.3 | Skin/subcutaneous/muscle [except rectus abdominus] | Avulsion blood loss >20% by volume | 510806.3 | 510806.3 | 5 |
| 520208.5 | Aorta, Abdominal | Laceration; perforation; puncture: major; rupture; transection; segmental loss: blood loss >20% by volume | 520208.5 | 520208.5 | 5 |
| 520408.5 | Celiac Artery | Laceration; perforation; puncture major; rupture; transection; segmental loss: blood loss >20% by volume | 520408.5 | 520408.5 | 5 |
| 520608.4 | Iliac Artery [common, internal, external] and its named branches | Laceration; perforation; puncture major; rupture; transection; segmental loss: blood loss >20% by volume | 520608.4 | 520608.4 | 5 |
| 521108.4 | Superior Mesenteric Artery | Laceration; perforation; puncture major; rupture; transection; segmental loss; blood loss >20% by volume | 521408.4 | None | 5 |
| 521408.4 | Other named arteries | Laceration; perforation; puncture major; rupture; transection; segmental loss: blood loss >20% by volume | 521408.4 | 521408.4 | 5 |
| 520806.4 | iliac Vein [common] | Laceration; perforation; puncture major; rupture; transection; segmental loss; blood loss >20% by volume | 520806.4 | 520806.4 | 4 |
| 521006.3 | Iliac Vein [internal, external] | Laceration; perforation; puncture major; rupture; transection; segmental loss: blood loss >20% by volume | 521006.3 | 521006.3 | 4 |
| 521206.4 | Vena Cava, Inferior | Major; rupture; transection; segmental loss; blood loss >20% by volume | 521206.4 | 521206.4 | 5 |
| 521606.4 | Other named veins | Laceration; perforation; puncture major; rupture; transection; segmental loss; blood loss >20% by volume | 521606.4 | 521606.4 | 5 |
| 541626.4 | Kidney | Laceration extending through renal cortex, medulla and collecting system; main renal vessel injury with hemorrhage; major [OIS IV] | 541626.4 | 541626.4 | 5 |
| 541628.5 | Kidney | Laceration hilum avulsion; total destruction of organ and its vascular system [OIS V] | 541628.5 | 541628.5 | 5 |
| 541826.4 | Liver | Laceration parenchymal disruption 75% hepatic lobe; multiple lacerations >3cm deep; burst injury: major [OIS IV] | 541826.4 | 541826.4 | 5 |
| 541828.5 | Liver | Laceration parenchymal disruption of >75% of hepatic lobe or >3 Coulnard’s segments within a single lobe; or involving retro hepatic vena cava/ central hepatic veins; massive; complex [OIS V] | 541828.5 | 541828.5 | 5 |
| 541830.6 | Liver | Laceration hepatic avulsion (total separation of all vascular attachments) [OIS VI] | 541830.6 | 541830.6 | 1 |
| 544226.4 | Spleen | Laceration involving segmental or hilar vessels producing major devascularization of >25% of spleen but no hilar injury; major [OIS IV] | 544226.4 | 544226.4 | 5 |
| 544228.5 | Spleen | Laceration hilar disruption producing total devascularization; tissue loss; avulsion; massive [OIS V] | 544228.5 | 544228.5 | 5 |
| 711000.3 | Amputation [traumatic], partial or complete | Between shoulder and hand. but NFS as to specific anatomical site | 711000.3 | 711000.3 | 2 |
| 711001.4 | Amputation [traumatic], partial or complete | At shoulder | 711000.3 | None | 1 |
| 711010.5 | Amputation [traumatic], partial or complete | At shoulder bilateral | 711000.3 | None | 1 |
| 711002.4 | Amputation [traumatic], partial or complete | At or above elbow, below shoulder | 711000.3 | None | 1 |
| 711012.5 | Amputation [traumatic], partial or complete | At or above elbow, below shoulder bilateral | 711000.3 | None | 1 |
| 711003.3 | Amputation [traumatic], partial or complete | Below elbow, at or above wrist | 711000.3 | None | 1 |
| 716006.3 | Penetrating injury | NFS as to specific anatomical sites with blood loss >20% by volume | 716006.3 | 716006.3 | 5 |
| 716013.3 | Penetrating injury | At shoulder, with blood loss >20% by volume | 716006.3 | None | 5 |
| 716017.3 | Penetrating injury | At or above elbow, below shoulder, with blood loss >20% by volume | 716006.3 | None | 5 |
| 710606.3 | Skin/subcutaneous/muscle | Laceration blood loss >20% by volume | 710606.3 | 710606.3 | 5 |
| 710806.3 | Skin/subcutaneous/muscle | Avulsion blood loss >20% by volume | 710806.3 | 710806.3 | 5 |
| 720208.3 | Axillary artery | Laceration; perforation; puncture major; rupture; transection; segmental loss: blood loss >20% by volume | 720208.3 | 720208.3 | 5 |
| 720406.3 | Axillary vein | Laceration; perforation; puncture major; rupture; transaction: segmental loss; blood loss >20% by volume | 720406.3 | 720406.3 | 5 |
| 720608.3 | Brachial artery | Laceration; puncture: perforation major; rupture; transection; segmental loss; blood loss >20% by volume | 720608.3 | 720608.3 | 5 |
| 720806.3 | Brachial vein | Laceration; puncture: perforation major; rupture; transection; segmental loss; blood loss >20% by volume | 720806.3 | 720806.3 | 5 |
| 721008.3 | Other named arteries [e.g., radial, ulnar] | Laceration; puncture: perforation major; rupture; transection; segmental loss; blood loss >20% by volume | 721008.3 | 721008.3 | 5 |
| 721206.3 | Other named veins [e.g., cephalic, basilic] | Laceration; puncture: perforation major; rupture; transection; segmental loss; blood loss >20% by volume | 721206.3 | 721206.3 | 5 |
| 811000.3 | Amputation [traumatic], partial or complete | Between hip and foot. but NFS as to specific anatomical site | 811000.3 | 811000.3 | 2 |
| 811001.4 | Amputation [traumatic], partial or complete | At hip or at buttock | 811004.4 | 811004.4 | 1 |
| 811010.5 | Amputation [traumatic], partial or complete | At hip or at buttock bilateral | 811004.4 | None | 1 |
| 811002.4 | Amputation [traumatic], partial or complete | At or above knee, below hip | 811004.4 | 811004.4 | 2 |
| 811012.5 | Amputation [traumatic], partial or complete | At or above knee, below hip bilateral | 811004.4 | None | 1 |
| 811003.3 | Amputation [traumatic], partial or complete | Below knee, at or above ankle | 811002.3 | 811002.3 | 2 |
| 816006.3 | Penetrating Injury | NFS as to specific anatomical site with blood loss >20% by volume | 816006.3 | 816006.3 | 4 |
| 816013.3 | Penetrating Injury | At hip or buttock, with blood loss >20% by volume | 816006.3 | None | 5 |
| 816017.3 | Penetrating Injury | At or above knee, below hip, blood loss >20% by volume | 816006.3 | None | 5 |
| 810606.3 | Skin/subcutaneous/muscle | Laceration blood loss >20% by volume | 810606.3 | 810606.3 | 5 |
| 810806.3 | Skin/subcutaneous/muscle | Avulsion blood loss >20% by volume | 810806.3 | 810806.3 | 5 |
| 820208.4 | Femoral artery and its named branches | Major; rupture; transection; segmental loss; blood loss >20% by volume | 820208.4 | 820208.4 | 5 |
| 820406.3 | Femoral vein | Laceration; perforation; puncture major: rupture; transection; segmental loss; blood loss >20% by volume | 820406.3 | 820406.3 | 4 |
| 820608.3 | Popliteal artery | Laceration; perforation; puncture major; rupture: transection; segmental loss; blood loss >20% by volume | 820608.3 | 820608.3 | 5 |
| 820806.3 | Popliteal vein | Laceration; perforation; puncture major; rupture; transection; segmental loss; blood loss >20% by volume | 820806.3 | 820806.3 | 4 |
| 821008.3 | Other named arteries [e.g., tibia, peroneal] | Laceration; perforation; puncture major; rupture; transection; segmental loss; blood loss >20% by volume | 821008.3 | 821008.3 | 5 |
| 821206.3 | Other named veins [e.g., saphenous] | Laceration; perforation; puncture major; rupture; transection; segmental loss; blood loss >20% by volume | 821206.3 | 821206.3 | 4 |
| 856164.5 | Pelvic ring | Fracture, incomplete disruption of posterior arch NFS blood loss > 20% by volume | 852610.5 | None |  |
| 856173.5 | Pelvic ring | Fracture, complete disruption of posterior arch and pelvic floor NFS blood loss > 20% by volume | 852610.5 | None |  |
