# Supplementary material for: Pre-hospital tranexamic acid administration in patients with a severe hemorrhage: an evaluation after the implementation of tranexamic acid administration in the Dutch pre-hospital protocol
Source: Eur J Trauma Emerg Surg. 2023 Apr 17;50(1):139–47. doi: 10.1007/s00068-023-02262-4 (PMC10923991; doi:10.1007/s00068-023-02262-4)
Supplement: Supplementary file 2 — Supplementary file2 (DOCX 14 KB) [file 68_2023_2262_MOESM2_ESM.docx]

| **Appendix 2.** Tranexamic acid treatment rate per ambulance service | | | | | | | | |
| --- | --- | --- | --- | --- | --- | --- | --- | --- |
| **Variables** | **Total**  n = 477 | **Service 1**  n = 135 | **Service 2**  n = 64 | **Service 3**  n = 94 | **Service 4**  n = 36 | **Service 5**  n = 80 | **Service 6**  n = 16 | **Service 7**  n = 52 |
|  | **N (%)** | **N (%)** | **N (%)** | **N (%)** | **N (%)** | **N (%)** | **N (%)** | **N (%)** |
| Tranexamic acid + | 124 (26.0) | 23 (17.0) | 32 (50.0) | 24 (25.5) | 16 (44.4) | 9 (11.3) | 8 (50.0) | 12 (23.1) |
| No tranexamic acid | 353 (74.0) | 112 (83.0) | 32 (50.0) | 70 (74.5) | 20 (55.6) | 71 (88.7) | 8 (50.0) | 40 (76.9) |
| Suspected hemorrhage* | 206 (58.4) | 58 (51.8) | 16 (50.0) | 45 (64.3) | 13 (65.0) | 52 (73.2) | 4 (50.0) | 18 (45.0) |
| *Hemorrhage suspected in non-treated patients | | | | | | | | |
