# Supplementary material for: Pre-hospital tranexamic acid administration in patients with a severe hemorrhage: an evaluation after the implementation of tranexamic acid administration in the Dutch pre-hospital protocol
Source: Eur J Trauma Emerg Surg. 2023 Apr 17;50(1):139–47. doi: 10.1007/s00068-023-02262-4 (PMC10923991; doi:10.1007/s00068-023-02262-4)
Supplement: Supplementary file 3 — Supplementary file3 (DOCX 14 KB) [file 68_2023_2262_MOESM3_ESM.docx]

| **Appendix 3.** Sensitivity analysis | | | |
| --- | --- | --- | --- |
| **Variables** | **Total**  n = 477 | **Patients without missing SBP**  n = 366 | **Patients without isolated grade ≥ 4 visceral organ injury**  n = 363 |
|  | **N (%)** | **N (%)** | **N (%)** |
| Tranexamic acid + | 124 (26.0) | 94 (25.7) | 106 (29.2) |
| No tranexamic acid | 353 (74.0) | 272 (74.3) | 257 (70.8) |
| Suspected hemorrhage* | 206 (58.4) | 157 (57.7) | 172 (66.9) |
| *Hemorrhage suspected in non-treated patients | | | |
