# Supplementary material for: Pre-hospital tranexamic acid administration in patients with a severe hemorrhage: an evaluation after the implementation of tranexamic acid administration in the Dutch pre-hospital protocol
Source: Eur J Trauma Emerg Surg. 2023 Apr 17;50(1):139–47. doi: 10.1007/s00068-023-02262-4 (PMC10923991; doi:10.1007/s00068-023-02262-4)
Supplement: Supplementary file 4 — Supplementary file4 (DOCX 16 KB) [file 68_2023_2262_MOESM4_ESM.docx]

| **Appendix 4.** Tranexamic acid treatment per body region | | | | | | | | | | | | | | | | |
| --- | --- | --- | --- | --- | --- | --- | --- | --- | --- | --- | --- | --- | --- | --- | --- | --- |
| **Variables** | **All patients with a severe hemorrhage**  n = 502 | | | | **Head, face, or neck involved**  n = 78 | | | **Thorax involved**  n = 74 | | | **Abdomen involved**  n = 210 | | | **Extremities involved**  n = 158 | | |
|  | **TXA +**  n = 124 | **TXA -**  n = 378 | ***p* value** | **TXA +**  n = 15 | | **TXA -**  n = 63 | ***p* value** | **TXA +**  n = 23 | **TXA -**  n = 51 | ***p* value** | **TXA +**  n = 56 | **TXA -**  n = 154 | ***p* value** | **TXA +**  n = 39 | **TXA -**  n = 119 | ***p* value** |
| **Pre-hospital vitals** | **N (%)** | **N (%)** |  | **N (%)** | | **N (%)** |  | **N (%)** | **N (%)** |  | **N (%)** | **N (%)** |  | **N (%)** | **N (%)** |  |
| Systolic blood pressure  <90 mmHg | 27 (21.8) | 39 (10.3) | 0.003 | 4 (26.7) | | 3 (4.8) | 0.034 | 5 (21.7) | 8 (15.7) | 0.814 | 9 (16.1) | 9 (5.8) | 0.040 | 13 (33.3) | 18 (15.1) | 0.043 |
| Heart rate >110 bpm | 48 (38.7) | 75 (19.8) | <0.001 | 1 (6.7) | | 10 (15.9) | 0.770 | 10 (43.4) | 15 (29.4) | 0.396 | 26 (46.4) | 29 (18.8) | <0.001 | 16 (41.0) | 24 (20.2) | 0.017 |
| Respiratory rate  > 29/min or  < 10/min | 24 (19.4) | 37 (9.8) | 0.014 | 2 (13.3) | | 5 (7.9) | 0.700 | 8 (34.8) | 10 (19.6) | 0.383 | 11 (19.6) | 10 (6.5) | 0.018 | 6 (15.4) | 13 (10.9) | 0.070 |
| Glasgow Coma Scale  <13 | 56 (45.2) | 85 (22.5) | <0.001 | 8 (53.3) | | 18 (28.6) | 0.141 | 12 (52.2) | 23 (45.1) | 0.701 | 31 (55.4) | 25 (16.2) | <0.001 | 12 (30.8) | 27 (22.7) | 0.430 |
| Abbreviations: TXA, tranexamic acid  Patients can sustain severe hemorrhages in multiple body regions. All missing values were multiply imputed. | | | | | | | | | | | | | | | | |

Appendix 4.
